# Mycobacterial Interspersed Repeat Unit–Variable Number Tandem Repeat Typing of *Mycobacterium avium* Strains Isolated from the Lymph Nodes of Free-Living Carnivorous Animals in Poland

**DOI:** 10.3390/pathogens12091184

**Published:** 2023-09-21

**Authors:** Blanka Orłowska, Marta Majchrzak, Anna Didkowska, Krzysztof Anusz, Monika Krajewska-Wędzina, Anna Zabost, Sywia Brzezińska, Monika Kozińska, Ewa Augustynowicz-Kopeć, Kaja Urbańska, Mirosław Welz, Paweł Parniewski

**Affiliations:** 1 Department of Food Hygiene and Public Health Protection, Institute of Veterinary Medicine, Warsaw University of Life Sciences (SGGW), Nowoursynowska 159, 02-776 Warsaw, Poland; anna_didkowska@sggw.edu.pl (A.D.); krzysztof_anusz@sggw.edu.pl (K.A.); 2 Institute of Medical Biology, Polish Academy of Sciences, Lodowa 106, 93-232 Lodz, Poland; 3 Department of Microbiology, National Veterinary Research Institute, Aleja Partyzantów 57, 24-100 Puławy, Poland; kappa2@wp.pl; 4 Department of Microbiology, National Tuberculosis Reference Laboratory, National Tuberculosis and Lung Diseases Research Institute, Płocka 26, 01-138 Warsaw, Poland; a.zabost@igichp.edu.pl (A.Z.); s.brzezinska@igichp.edu.pl (S.B.); m.kozinska@igichp.edu.pl (M.K.); e.kopec@igichp.edu.pl (E.A.-K.); 5 Department of Morphological Sciences, Division of Histology and Embryology, Institute of Veterinary Medicine, Warsaw University of Life Sciences (SGGW), Nowoursynowska 159, 02-776 Warsaw, Poland; kaja_urbanska@sggw.edu.pl; 6 Provincial Veterinary Inspectorate, Piotra Ścigiennego 6a, 38-400 Krosno, Poland; m-welz@o2.pl

**Keywords:** free-living carnivores, MAC, MIRU-VNTR, *Mycobacterium avium* complex, non-tuberculous mycobacteria, NTM, Podkarpackie Voivodeship, the Bieszczady Mountains, wildlife

## Abstract

Non-tuberculous mycobacteria (NTM) are ubiquitous organisms, of which some, especially those of the *Mycobacterium avium* complex (MAC), may be opportunistic animal and human pathogens. Infection with NTM can interfere with tuberculosis (TB) diagnosis and induce zoonoses, especially in immunocompromised individuals. Diseases caused by NTM have become more readily recognized; however, they are likely still underestimated. In this study, we identified and genotyped *Mycobacterium avium* strains that were isolated during TB monitoring among free-living carnivorous animals from southeastern Poland. In 2011–2020, lymph node samples from 192 such animals were tested for mycobacteria. A total of 41 isolates of *M. avium* strains were detected with the use of IS901, IS900, IS1245, and mycobacterial interspersed repeat unit–variable number tandem repeat (MIRU-VNTR) identification. Thirty-three were identified as *M. avium* subsp. *avium.* These strains were derived from 1 beech marten (*Martes foina*), 1 common buzzard (*Buteo buteo*), 2 European badgers (*Meles meles*), 3 wolves (*Canis lupus*), and 26 red foxes (*Vulpes vulpes*). One strain isolated from a wolf was identified as *M. avium* subsp. *hominissuis*. The results show the widespread occurrence of MAC bacilli in the studied environment and additionally comprise new data on the molecular characteristics of *M. avium* subspecies carried by free-living southeastern Polish carnivores.

## 1. Introduction

Most non-tuberculous mycobacteria (NTM) are ubiquitous environmental organisms [1]. However, some of them, especially those of the *Mycobacterium avium* complex (MAC), are opportunistic pathogens [2,3,4,5,6] and may cause lymphadenitis and infections of the lungs, skin, soft tissue, and bones, among other disorders [2,7,8]. One of the species belonging to MAC is *Mycobacterium avium*. *M. avium* is divided into four subspecies: *avium* (MAA), *silvaticum* (MAS), *hominissuis* (MAH), and *paratuberculosis* (MAP) [9]. Among all NTM, MAC members are the bacteria most commonly associated with pulmonary disease in humans [10].

Infection with NTM can interfere with tuberculosis (TB) diagnosis [11,12] and carries some risk of zoonoses, especially in immunocompromised individuals [13,14,15]. Non-tuberculous mycobacteria have been found in many wildlife species [16], such as the badger (*Meles meles*) [17], wild boar (*Sus scrofa*) [18], fallow deer (*Dama dama*) [19], otter (*Lutra lutra*) [20], wild rabbit (*Oryctolagus cuniculus*) [21], roe deer (*Capreolus capreolus*) [22], red deer (*Cervus elaphus*), sika deer (*Cervus nippon*) [23], and various species of wild rodents [24]. Diseases caused by NTM have become more readily recognized; however, it seems that they are still underestimated [7].

Molecular analysis of NTM strains isolated from wildlife is rarely conducted [25] and according to our best knowledge, has never been undertaken in Poland. To date, the only described cases of NTM infection in wildlife in Poland occurred in three European bison (*Bison bonasus*), from which *Mycobacterium xenopi* and *Mycobacterium avium* were isolated [26], and in one wild boar, from which *Mycobacterium kansasii* complex was isolated [27]. In Poland, the occurrence of NTM was also studied in domesticated animals, namely pigs. *Mycobacterium avium* complex members were isolated, including *M. avium* subsp. *paratuberculosis* (MAP) [28].

NTM can be identified by genotyping, which is a valuable method, especially regarding epidemiological investigations. It can be helpful in finding both the source of infection and the possible routes of transmission. One of the most commonly used methods for genotyping MAC is the mycobacterial identification repetitive unit–variable number of tandem repeats (MIRU-VNTR) method [29], which is based on eight loci with highly discriminated indices: MIRU 292, MIRU X3, VNTR 25, VNTR 47, VNTR 3, VNTR 7, VNTR 10, and VNTR 32 [30]. Insertion sequence (IS) analysis is also feasible [28]. This genotyping method was selected for the present study because in addition to its usefulness in determining the types of isolated strains, it can show which types are dominant and whether they coincide with the types found with the IS method in livestock in a previous study [28]. Identical MIRU-VNTR patterns may indicate a risk of strain transmission between wild animals and livestock. Carnivores are appropriate wild animals to investigate because, being at the top of the food chain, they can indicate the presence of a given pathogen in the environment [31].

The aim of this study was to, for the first time in Poland, identify and genotype *Mycobacterium avium* strains that were collected and isolated during TB monitoring of carnivorous free-living animals in the Podkarpackie Voivodeship (southeastern Poland). Previously, TB was confirmed in wildlife in the Podkarpackie Voivodeship [32,33,34].

## 2. Materials and Methods

### 2.1. Materials

Materials from 192 free-living animals were analyzed collaterally for the presence of tuberculosis. The samples were collected from 141 red foxes (*Vulpes vulpes*), 27 wolves (*Canis lupus*), 11 badgers (*Meles meles*), 5 Eurasian lynxes (*Lynx lynx*), 4 European wildcats (*Felis silvestris*), 1 beech marten (*Martes foina*), 1 buzzard (*Buteo buteo*), 1 brown bear (*Ursus arctos*), and 1 raccoon dog (*Nyctereutes procyonoides*). The materials were collected in the Podkarpackie Voivodeship in southeastern Poland from 2011 to 2020. Forty-one isolates of *M. avium* strains were identified in this study.

The studied foxes, martens, raccoons, and badgers were shot during the hunting season and delivered by hunters to the Veterinary Hygiene Institution (VHI) in Krosno (Poland) as part of the procedure for rabies surveillance. The samples used in this study had to be free from the rabies virus. Materials from wolves, buzzards, brown bears, wildcats, and lynxes were collected from animals found dead (most of them fatally struck by vehicles). Some wolves had been culled because of their bold behavior and potential danger to humans or livestock. The shooting was carried out with the permission of the General Director for Environmental Protection. Official Veterinarians of the Provincial Veterinary Inspectorate performed all necropsies and sample collections. The samples taken from the foxes, badgers, beech marten, and raccoon dog by the VIH comprised mandibular and mesenteric lymph nodes. The samples from the remaining animals comprised the mandibular, retropharyngeal, and mesenteric lymph nodes. The investigated samples from the buzzard consisted of internal organs and lymph nodes. The samples were transported to the laboratory under refrigeration. All collected lymph nodes were cut into 1.0 to 1.5 mm thick slices and macroscopically examined for the presence of lesions. Lymph nodes from each individual were pooled and microbiologically tested to detect mycobacteria. The buzzard’s organs were examined and tested in the same manner.

### 2.2. Culture

The culture procedure was performed according to the recommendations of the Reference Microbiological Laboratory of the National Veterinary Research Institute in Puławy, Poland [35]. Briefly, the material was minced with scissors, decontaminated with a 5% oxalic acid solution, and homogenized. The sediment was plated onto two solid media: Stonebrink (Becton Dickinson, Franklin Lakes, NJ, USA) and Löwenstein–Jensen (Becton Dickinson). The media were incubated at 37 °C for 12 weeks. Growth was assessed once a week. Rough white-to-yellow colonies were considered *Mycobacterium* sp., and no growth after 12 weeks was considered a negative result.

The isolated mycobacteria that were not *Mycobacterium tuberculosis* complex (MTBC) species were typed using the MIRU-VNTR method and checked for IS type as described in a previous study [28].

### 2.3. DNA Isolation

The thermal method was used to extract DNA from colonies grown on the Löwenstein–Jensen and Stonebrink media. Briefly, the colony was suspended in 150 μL of water and then incubated for 30 min in a thermoblock at 95 °C. After incubation, the tubes were centrifuged for 5 min at 15,000× *g*. The supernatant was used for testing.

### 2.4. Strain Identification

The isolated strains were identified using a GenoType^®^ Mycobacterium CM (“Common Mycobacteria”) test (Hain Lifescience, Nehren, Germany) performed according to the manufacturer’s instructions. Non-tuberculous mycobacteria were further identified as described below.

### 2.5. IS901, IS900, and IS1245 Identification

Forty-one isolates were subjected to IS901, IS900, and IS1245 analysis according to the method previously referred to with modifications [28]. This method is used for the rapid identification of *M. avium* subspecies. For IS900, IS901, and IS1245, the PCR reaction was performed in a total volume of 50 µL containing the following components: 20 ng of DNA, 25 µL of Platinum Multiplex PCR Master Mix 2× (Applied Biosystems, Carlsbad, CA, USA), and 50 nM of each primer. The following conditions were used: an initial denaturation step at 95 °C for 15 min; 28 cycles of denaturation at 95 °C for 30 s, annealing at 60 °C for 90 s, and extension at 72 °C for 1 min; and a final extension step at 72 °C for 2 min. All reactions were performed using a T3000 thermal cycler (Analytik Jena, Jena, Germany).

Electrophoresis was performed at 70 V (2.4 V/cm) until the bromophenol blue dye reached 6 cm from the wells. The gels were then stained in an ethidium bromide solution (0.5 µg/mL) for 10 min and destained in water for another 10 min. The gels were visualized under UV light using a FluorChem 8800 system with Alpha EaseFC v. 3.1.2 software (AlphaInnotech, San Leandro, CA, USA). A 100 bp Plus ladder size marker from MBI Fermentas (Vilnius, Lithuania) was used for all analyzed IS types. The predicted sizes of the PCR fragments for IS1245, IS900, and IS901 were 427 bp, 389 bp, and 262 bp, respectively.

### 2.6. MIRU-VNTR Identification

The MIRU-VNTR method used in the present study is a simple tool for genotyping mycobacteria and detecting possible phylogenetic relationships between strains. The isolates were subjected to multi-locus variable-number tandem-repeat analysis (MLVA) according to the eight variable-number tandem-repeat loci scheme proposed by Thibault et al. [30]. Primer sequences for the amplification of the TR292, TRX3, TR25, TR47, TR3, TR7, TR10, and TR32 loci were selected from Cochard et al. [36], and the PCR was optimized and performed as previously described [28].

The size of each amplicon was measured with BioNumerics software version 4.6 (Applied Maths, Sint-Martens-Latem, Belgium) and used to assess the number of motif repeats. The numerical codes were compared with those registered in the MAC-INMV-SSR database (http://mac-inmv.tours.inra.fr/ accessed on 28 February 2023 and 24 August 2023).

## 3. Results

### 3.1. Post-Mortem Examination

Tuberculous lesions were detected only in the internal organs of the buzzard. Whitish-yellow caseous nodules 1–2 mm in diameter were present on the stomach, liver, and intestines. The remaining tested animals’ samples (lymph nodes) had no visible lesions. Most of the examined animals were shot during the hunting season or were road kill.

### 3.2. Mycobacterial Analysis and Species Designation

The isolated strains were classified as *M. avium* based on the appearance of single colonies on the differentiating media. To confirm the presence of MAC strains, IS analysis was performed. Out of 41 strains, 36 showed the presence of IS901, 34 showed the presence of IS1245, and none showed the presence of IS900 (Table 1).

Thirty-three of the studied strains were identified as MAA. These strains were isolated from the material from 33 free-living animals: 1 beech marten (*Martes foina*), 1 common buzzard (*Buteo buteo*), 2 European badgers (*Meles meles*), 3 wolves (Canis lupus), and 26 red foxes (*Vulpes vulpes*). These strains showed the presence of IS901 and IS1245 and the absence of IS900.

One strain isolated from a wolf (isolate W32) was identified as MAH. This strain lacked IS901 and IS900 but contained IS1245.

Seven strains isolated from one badger, two wolves, and four foxes were identified as not being *M. avium* spp. Four of these strains had no IS, and three only showed the presence of IS901.

### 3.3. MIRU-VNTR Analysis

Nine MIRU-VNTR patterns were identified in 33 tested isolates (Table 1). Five numerical codes (12131227, 13131227, 25131237, 33131227, and 25321228) were not identified; they were added to the MAC-INMV-SSR database. No MLVA patterns were identified for four cases, and in three cases, the MLVA pattern was incomplete.

**Figure 1:**
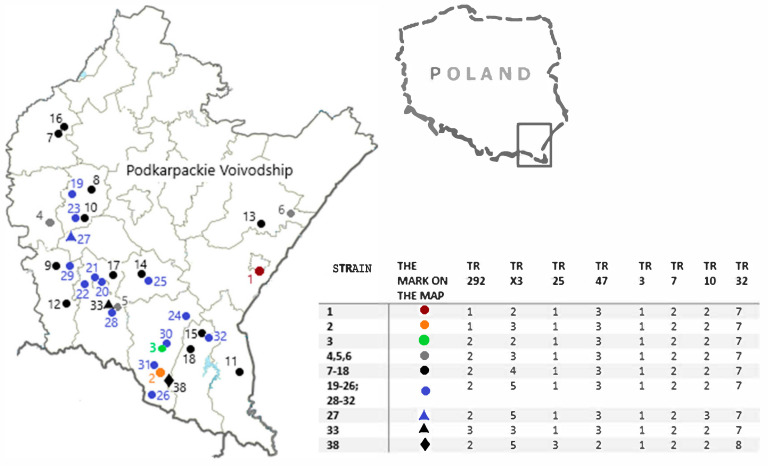
The geographical origin of the 34 MIRU-VNTR *Mycobacterium avium* profiles included in the study.

The results of the molecular analysis (which are the MIRU-VNTR patterns and the presence of IS901, IS900, and IS1245) and their geographical distribution are presented in Table 1 and Figure 1.

## 4. Discussion

Our research presents new data on the molecular characteristics of *M. avium* subspecies isolated from free-living animals in the Podkarpackie Voivodeship of southeastern Poland. These are the first studies in Poland on the occurrence of *M.avium* in free-living animals using the MIRU-VNTR technique. The studied strains were isolated from red foxes, grey wolves, badgers, pine marten, and common buzzard.

Generalized mycobacteriosis was found in a buzzard. The lesions mainly occurred in the liver, stomach, and intestines. The *M. avium* spp. *avium* is considered an obligate pathogen of birds, and many wild and domesticated bird species are susceptible to MAA infection and develop the clinical form of the disease [37,38].

The isolated MAA and MAH strains did not cause any visible macroscopic lesions in the tested group of mammals (in the examined tissues of other animals, i.e., foxes, badgers, wolves, and martens). In the cases of foxes and wolves, these observations are similar to those concerning MTBC infections in these species. In predatory species such as foxes, wolves, and coyotes, macroscopic changes were not often observed despite MTBC isolation. Tuberculous lesions, if present, were often found in the mesenteric lymph nodes [39,40,41,42,43].

Mycobacteriosis is found in various wild animals [44,45,46] and domesticated animals, e.g., birds [47,48], cattle [49], and horses [50]. Schrenzel et al. [51] found that *M. avium* ssp. *avium* in birds’ feces may be the source of environmental contamination with these mycobacteria. Durnez et al. [52,53] isolated various mycobacteria, including MAC, from rodents and small insectivorous mammals. *Mycobacterium avium* complex members were also isolated from soil and water [54,55] and from earthworms [56]. Bacteria of this complex are also found in raw meat, which may be a source of infection [57].

In our research, *M. avium* ssp. were isolated from carnivorous animals (32 mammals and 1 bird). One of the species that carries these bacteria, the badger, has close contact with the ground because of its lifestyle, and its favorite food is earthworms. It is possible that the tested animals became infected by eating mycobacteria- or soil-contaminated tissues of other animals or by drinking contaminated water.

Interestingly, there was no case of simultaneous isolation of NTM and MTBC. In the tested animals, either NTM or MTBC were isolated, but never both from the same sample. Nevertheless, caution is advised in attributing any pattern to this finding from the study described in this article because, in the present sample set of 192 free-living carnivorous animals analyzed during the conduct of a tuberculosis monitoring program, MTBC was isolated from only three wolves [33]. In this research, we used solid media for the isolation of mycobacteria, which may be less sensitive than culture in liquid media. Infection of Podkarpackie Voivodeship fauna with *M. avium* was previously found in red deer and wild boar. However, their MIRU-VNTR patterns were not studied [58,59].

In our study, a total of 33 of the studied strains were identified as MAA [60]. The results of the investigation of these 33 strains showed that three of the eight studied loci (TR 292, TR x3, and TR 10) were polymorphic, while the others (TR 25, TR 47, TR 3, TR 7, and TR 32) had the same repeat count in all tested strains. One strain isolated from a wolf (W32) was identified as MAH [60]. This strain lacked IS901 and IS900 but carried IS1245. Its MIRU-VNTR pattern was polymorphic in five loci (MIRU-VNTR; 25321228), whereas three loci were polymorphic in the 33 strains identified as *M. avium* spp. in the present investigation. Five new MIRU-VNTR patterns (12131227, 13131227, 25131237, 33131227, and 25321228) were added to the MAC-INMV-SSR database.

Seven strains were identified as not *M. avium* spp. For three of these strains (isolates: 134, W18, and W15), it was impossible to obtain all MIRU-VNTR patterns, but the presence of IS901 may suggest that they could have been *M. avium* spp. In such cases, other tests should be performed, including identification by sequencing of 16S rRNA, rpoB, or hsp65, or by MALDI-TOF or WGS. However, this research did not focus on such deep subtyping of atypical mycobacteria isolated from free-living animals.

It is worth emphasizing that most of the MIRU-VNTR types identified by us in these studies coincide with those previously found in pigs in another region of Poland [28]. MIRU-VNTR types such as 22131227, 23131227, 24131227, and 25131227 found in pigs were also identified in our study (one buzzard, one badger, two wolves, and twenty-five foxes—Table 1). In our opinion, this is an interesting observation, and the transmission of atypical mycobacteria between farm animals and free-living animals should be further investigated.

## 5. Conclusions

The results of this study showed the widespread occurrence of MAC bacilli in the studied environment. It is difficult to assess what role MAAs play in the examined animals, especially since no pathological lesions were found in the examined mammals. MAA species are opportunistic pathogens for mammals, including humans, which are of particular importance in immunocompromised individuals [2]. Cases of diseases caused by atypical mycobacteria in humans are becoming more frequent [61,62,63,64,65]. Human contact with wild animals is not as frequent and intense as with companion animals, but certain groups of people, such as hunters, wildlife researchers, and veterinarians, may be more exposed to wild animal pathogens. In addition, infection with atypical mycobacteria may affect tuberculin test results [66] and tests for paratuberculosis [67].

## Figures and Tables

**Table 1:** Results of MIRU-VNTR typing and IS901, IS900, and IS1245 identification of 41 *M. avium* strains isolated from free-living animals from southeastern Poland.

No. (Isolate Number)	The Origin of the Strain	Number of Copies of MIRU-VNTR Region								Subspecies Assignment	IS901	IS900	IS1245
		TR 292	TR x3	TR 25	TR 47	TR 3	TR 7	TR 10	TR 32				
1 (169)	Beech marten	1	2	1	3	1	2	2	7	MAA	+	-	+
2 (23 b)	European badger	1	3	1	3	1	2	2	7	MAA	+	-	+
3 (W17)	Grey wolf	2	2	1	3	1	2	2	7	MAA	+	-	+
4 (603)	Red fox	2	3	1	3	1	2	2	7	MAA	+	-	+
5 (493)	Red fox	2	3	1	3	1	2	2	7	MAA	+	-	+
6 (494)	Red fox	2	3	1	3	1	2	2	7	MAA	+	-	+
7 (522)	Red fox	2	4	1	3	1	2	2	7	MAA	+	-	+
8 (56)	Red fox	2	4	1	3	1	2	2	7	MAA	+	-	+
9 (108)	Red fox	2	4	1	3	1	2	2	7	MAA	+	-	+
10 (579)	Red fox	2	4	1	3	1	2	2	7	MAA	+	-	+
11 (615)	Red fox	2	4	1	3	1	2	2	7	MAA	+	-	+
12 (619)	Red fox	2	4	1	3	1	2	2	7	MAA	+	-	+
13 (582)	Red fox	2	4	1	3	1	2	2	7	MAA	+	-	+
14 (517)	Red fox	2	4	1	3	1	2	2	7	MAA	+	-	+
15 (593)	Red fox	2	4	1	3	1	2	2	7	MAA	+	-	+
16 (523)	Red fox	2	4	1	3	1	2	2	7	MAA	+	-	+
17 (629)	Red fox	2	4	1	3	1	2	2	7	MAA	+	-	+
18 (488)	Red fox	2	4	1	3	1	2	2	7	MAA	+	-	+
19 (513)	Red fox	2	5	1	3	1	2	2	7	MAA	+	-	+
20 (510)	Red fox	2	5	1	3	1	2	2	7	MAA	+	-	+
21 (509)	Red fox	2	5	1	3	1	2	2	7	MAA	+	-	+
22 (498)	Red fox	2	5	1	3	1	2	2	7	MAA	+	-	+
23 (512)	Red fox	2	5	1	3	1	2	2	7	MAA	+	-	+
24 (461)	Red fox	2	5	1	3	1	2	2	7	MAA	+	-	+
25 (130)	Red fox	2	5	1	3	1	2	2	7	MAA	+	-	+
26 (1.4.5)	European badger	2	5	1	3	1	2	2	7	MAA	+	-	+
27 (529)	Red fox	2	5	1	3	1	2	3	7	MAA	+	-	+
28 (1 W)	Grey wolf	2	5	1	3	1	2	2	7	MAA	+	-	+
29 (171)	Red fox	2	5	1	3	1	2	2	7	MAA	+	-	+
30 (218)	Red fox	2	5	1	3	1	2	2	7	MAA	+	-	+
31 (Pt3)	Buzzard	2	5	1	3	1	2	2	7	MAA	+	-	+
32 (178)	Red fox	2	5	1	3	1	2	2	7	MAA	+	-	+
33 (W20)	Grey wolf	3	3	1	3	1	2	2	7	MAA	+	-	+
34 (520)	Red fox	-	-	-	-	-	-	-	-	NA	-	-	-
35 (83.4)	Red fox	-	-	-	-	-	-	-	-	NA	-	-	-
36 (447)	Red fox	-	-	-	-	-	-	-	-	NA	-	-	-
37 (235)	Red fox	-	-	-	-	-	-	-	-	NA	-	-	-
38 (W32)	Grey wolf	2	5	3	2	1	2	2	8	MAH	-	-	+
39 (W18)	Grey wolf	2	2	1	3	1	2	-	7	NA	+	-	-
40 (134)	European badger	2	5	1	3	1	2	-	-	NA	+	-	-
41 (W15)	Grey wolf	-	-	1	3	1	2	-	7	NA	+	-	-

MAA—*M. avium* ssp. *avium*; MAH—*M. avium* ssp. *hominisuis*; NA—not *M. avium*; “+”—presence of the tested IS; “-”—lack of the tested IS.

## Data Availability

Not applicable.
